# Effective and Selective Extraction of Quercetin from Onion (*Allium cepa* L.) Skin Waste Using Water Dilutions of Acid-Based Deep Eutectic Solvents

**DOI:** 10.3390/ma14216465

**Published:** 2021-10-28

**Authors:** Matteo Ciardi, Federica Ianni, Roccaldo Sardella, Stefano Di Bona, Lina Cossignani, Raimondo Germani, Matteo Tiecco, Catia Clementi

**Affiliations:** 1 Department of Chemistry, Biology and Biotecnology, University of Perugia, Via Elce di Sotto 8, 06123 Perugia, Italy; matteo.ciardi95@gmail.com (M.C.); dibonastefano@gmail.com (S.D.B.); raimondo.germani@unipg.it (R.G.); catia.clementi@unipg.it (C.C.); 2 Department of Pharmaceutical Sciences, University of Perugia, Via Fabretti 48, 06123 Perugia, Italy; federica.ianni@unipg.it (F.I.); roccaldo.sardella@unipg.it (R.S.); lina.cossignani@unipg.it (L.C.); 3 Center for Perinatal and Reproductive Medicine, University of Perugia, Santa Maria della Misericordia University Hospital, 06132 Perugia, Italy

**Keywords:** quercetin, Deep Eutectic Solvents, extraction, preconcentration, DESs water dilutions, RP-HPLC-UV, UHPLC-MS/MS, recovery

## Abstract

Deep Eutectic Solvents (DESs) are experiencing growing interest as substitutes of polluting organic solvents for their low or absent toxicity and volatility. Moreover, they can be formed with natural bioavailable and biodegradable molecules; they are synthesized in absence of hazardous solvents. DESs are, inter alia, successfully used for the extraction/preconcentration of biofunctional molecules from complex vegetal matrices. Onion skin is a highly abundant waste material which represents a reservoir of molecules endowed with valuable biological properties such as quercetin and its glycosylated forms. An efficient extraction of these molecules from dry onion skin from “Dorata di Parma” cultivar was obtained with water dilution of acid-based DESs. Glycolic acid (with betaine 2/1 molar ratio and L-Proline 3/1 molar ratio as counterparts) and of *p*-toluensulphonic acid (with benzyltrimethylammonium methanesulfonate 1/1 molar ratio)-based DESs exhibited more than 3-fold higher extraction efficiency than methanol (14.79 µg/mL, 18.56 µg/mL, 14.83 µg/mL vs. 5.84 µg/mL, respectively). The extracted quercetin was also recovered efficaciously (81% of recovery) from the original extraction mixture. The proposed extraction protocol revealed to be green, efficacious and selective for the extraction of quercetin from onion skin and it could be useful for the development of other extraction procedures from other biological matrixes.

## 1. Introduction

The substitution of common toxic and volatile organic solvents with novel greener liquids is of prior importance to tackle the urgent problems of planet pollution and improper chemical wastes disposal [1,2,3]. A large number of tons per year of volatile, toxic and bioaccumulating organic solvents are in fact used in chemical industries, playing the greatest part in chemical applications [4].

Deep Eutectic Solvents (DESs) are a novel class of organic liquids that are gaining increasing attraction in many sub-fields of chemical practice, as is well-documented by the recent literature [5,6,7,8]. A DES is an organic liquid endowed with valuable “green properties” which is formed via weak interactions, such as hydrogen bonds, between two (often solid) molecules, namely a hydrogen bond donor (HBD) and a hydrogen bond acceptor (HBA). The network of weak interactions established between the molecules of the same species and between the molecules of different species determines a difficult or even impossible crystal lattice formation. This ultimately leads to the formation of a liquid [9,10]. The resulting liquids show a deviation from ideal liquid mixtures in terms of the melting temperatures depending on the molar fraction of the components, with a deepening of the melting points as well as a shift in the molar ratio of the eutectic point [11]. Relevant papers are reported in literature with a quantitative approach to the phase diagrams of these liquids that can define these liquids as DESs or simple eutectic mixtures [12,13,14]. The preparation of these green mixtures represents a great step ahead in the formulation of innovative green liquids, especially whenever they are compared with other green liquids [15,16,17]. This is because the liquids are formed simply by heating and mixing the two often solid substances until homogenous systems are formed (often in few minutes) without the use of any other solvent. As a result, the realization processes, which often require only a few minutes, have 100% yield and 100% atom economy [5]. Many different molecules can be used to realize DESs liquids, including, but not limited to: onium salts with metal chlorides (also hydrated); choline chloride mixed with hydrogen bond donors (such as carboxylic acids); Lewis bases mixed with alcohols or amides; etc. [17,18,19,20].

The green properties of DESs rely on the fact that they generally (i) are non-toxic, (ii) have low or absent volatility (leading to the possibility of “out of the hood” procedures), (iii) are biodegradable and, (iv) in the case of natural source molecules used for their preparation (NADESs: Natural Deep Eutectic Solvents), their impact on the environment is markedly reduced, both in terms of bioavailability and biodegradability of the liquids themselves [21,22,23,24,25]. However, because the properties of the liquids’ components are retained in the properties of the DESs, not all these novel liquids can be considered of course as totally green as they can be formed by harmful or not green molecules. This is the case i.e., for highly acidic DESs components as *p*-toluenesulfonic acid. In these cases, the use of these liquids can nevertheless permit the avoidance of volatile mineral acidic components [26].

For the same reasons of the properties of the constituting components, DESs can also exhibit appreciable catalytic properties [8,27,28,29,30,31,32]. The effect seems to depend on the “availability” of the molecule forming the DES in exerting the catalytic action [33,34,35].

Another interesting facet about DESs, which is steadily gaining ground in the literature, is represented by the water dilutions of these weak forces-based systems, as water molecules can participate in the network of weak interaction [36,37]. The increase of the water dilutions leads to a solvation of clusters of couples of HBD-HBA molecules and to micro-domains of DESs and water; at values over about 50–60% *w*/*w* the DESs’ deconstruction occurs. Water dilutions, even after low amounts of added water, have peculiar and interesting physical-chemical properties as they show a high decrease of their viscosity and changes in their polarity [38]. These effects are however dependent on the hydrophobicity and hydrophilicity of the DESs’ components, so the values of water needed to determine structural changes can slightly shift.

Based on the last property, DESs are fruitfully applied in extraction and preconcentration procedures from different matrices, encompassing vegetal and biological ones [39,40]. One of the most interesting areas where DESs are finding relevant results is in their use as green liquids for biomass feedstocks treatments, where they are finding high effectiveness [41,42,43,44,45].

In particular, phenolic compounds of vegetal origin are successfully extracted/preconcentrated as they can participate in the hydrogen bonds network with the hydroxyl function as well as with the aromatic portions that can act as hydrogen bond acceptors [24,46].

Among the several naturally occurring flavonoids, quercetin is currently one of the most extensively studied because, besides its proven anti-blood clotting, cardioprotective, neuroprotective, anti-inflammatory, anti-cancer and antioxidant properties [47,48], it also shows an effective antiviral and immunomodulatory activity. In particular, recent studies suggest the efficacy of quercetin-based formulations in reducing symptoms severity and negative predictors of severe acute respiratory syndrome coronavirus 2 (SARS-CoV-2), which is the cause of the present COVID-19 global pandemic [49,50]. However, further studies are still needed to demonstrate these relevant data [51].

Among the different methods available for the extraction of quercetin, and of flavonoids from natural sources more generally, the ones based on ultrasound- and microwave-assisted procedures are the most widely applied. Besides the major well-recognized advantages of these techniques (fast execution, a certain level of environmentally friendly character, easy automation, etc.), some of their severe limitations and major drawbacks have been also described [52]. For example, thermal degradation of the compounds of interest, as well as their accidental participation in unwanted/uncontrolled side reactions are worthy of noting. Still, the use of even small percentages of volatile organic extraction solvents can represent a problem in terms of their environmental and safety impact. Equally important, using ultrasound- and microwave-assisted procedures implies a proper tuning and combination of several process variables, which must be cautiously optimized.

Scientific works describing the use of DESs and their water dilutions for the effective quercetin extraction from onion skin waste are already present in the literature [53]; the authors reported the use of common DESs such as choline chloride/urea/water mixtures or sugar-based DESs. Furthermore, applications regarding the efficient extraction performances of high water dilutions of DESs have been described [54].

Quercetin is contained in abundance in different varieties of vegetables and fruits such as apples, honey, raspberries, onions, red grapes, cherries, citrus fruits, and green and red leafy vegetables [55]. Among them, high quercetin content is found in yellow onion skin. Onion is one of the most important horticultural crops, which has reached a current worldwide production of around 100 million tons in 2019 leading to a consequent generation of a consistent amount of solid waste material. Recent literature reports that the annual European production of onion waste is around 500,000 tons, especially in major producing countries such as Spain, the Netherlands and the United Kingdom [56]. Onion skin, the most highly abundant waste material derived from onion processing, represents a reservoir of molecules endowed with valuable biofunctional properties [57,58]. Within the (phyto)complex, quercetin and in its glycosylated forms occupy a prominent position in this regard [59,60].

In this work, we present an effective and green procedure for the extraction of quercetin and its principal glycosylated form from dry onion skin of “Dorata di Parma” cultivar with the use of water dilutions of acidic DESs with a heating/stirring- and ultrasound-assisted protocol. This procedure revealed to be much more effective than the use of neat methanol, a protic highly toxic and volatile solvent commonly used in the extractions of polyphenols from complex matrixes [61,62]. An anti-solvent and a reversed-phase chromatography approach were performed to enable the raw quercetin recovery from each extract. In order to select the best parameters to maximize the recovery of quercetin(s) from onion extracts, the extraction efficiency was monitored by reversed phase-high performance liquid chromatography coupled to UV detection (RP-HPLC-UV). Ultra-high performance liquid chromatography-tandem mass spectrometry (UHPLC-MS/MS) analyses were also performed to allow the identification of the main peaks.

## 2. Materials and Methods

### 2.1. Reagents and Instruments

Glycolic acid (GA), Trimethylglycine (TMG), Ethylene Glycol (EG), Choline Chloride (ChCl), Glycerol (GLY), Urea (U), *p*-toluenesulfonic acid (pTSA), L-proline (L-PRO), Octanoic Acid (OCT), Decanoic Acid (DEC), Thymol (THY), Phenylacetic acid (PhAA), Methanol, Ethanol were purchased from Merck (Darmstadt, Germany) and Alfa-Aesar (Haverhill, MA, USA) and were used without further purifications (purities > 98.5%). Hygroscopic reagents were desiccated under P_2_O_5_ prior use. Trimethylbenzylammonium methanesulfonate was synthesized following a procedure reported elsewhere [26]. Water was used at milliQ purity grade (>18 MΩ).

A Sartorius LE225D was used as analytical balance; the centrifugations were performed using a Beckmann Coulter ALLEGRA 64R Centrifuge; Agilent 8453 UV-VIS Spectroscopy system equipped with a thermostat (25.0 ± 0.1 °C) was used for the UV-VIS spectra determination.

HPLC-grade and MS-grade acetonitrile (ACN, purity > 99.9%) and formic acid (FA purity ≥ 95%) were purchased from Sigma Aldrich (Milan, Italy). Water for HPLC analysis was purified with a Milli-Q Plus185 system from Millipore (Milford, MA, USA). The HPLC-UV study was performed on a Thermo Separation low-pressure quaternary gradient pump system (Spectra system Series, Thermo Scientific, Waltham, MA, USA) supplied with a GT-154 vacuum degasser (Shimadzu, Kyoto, Japan). The system was equipped with a SPD-10A UV-Vis detector (Shimadzu, Kyoto, Japan) and a Rheodyne 7725i injector (Rheodyne Inc., Cotati, CA, USA) with a 20 μL stainless steel loop. Data management and acquisition was made by means of Clarity Lite chromatography software. UV detection was carried out at 360 nm. A Robusta RP18 (250 × 4.6 mm i.d., 5 μm, 100 Å pore size from Sepachrom, Milan, Italy) was used as analytical column. A Grace (Sedriano, Italy) heater/chiller (Model 7956R) thermostat was used to carry out the RP-HPLC analyses at a column temperature fixed at 25 °C. All the analyses were carried out at a 1.0 mL min^−1^ flow rate. For UHPLC-MS/MS analysis an Agilent 1290 Infinity LC system coupled with an Agilent 6540 UHD Accurate Mass QTOF (Agilent Technologies, Santa Clara, CA, USA) with an Agilent Jet Stream Dual electrospray (Dual AJS ESI) interface was used. VELP Scientifica AREX oil bath with a VTF Vertex was used for the heating and the stirring of the samples, Branson BRANSONIC 220 sonicator bath (75 W sonication power) was used for the sonication procedure. The analytes separation was performed with a Kinetex (100 × 2.1 mm i.d., 1.7 µm, 100 Å) column from Phenomenex (Torrance, CA, USA) connected with a guard cartridge EVO-C18 (2.1 × 2 mm) from Phenomenex.

### 2.2. DESs Preparation and Water Dilutions

The Deep Eutectic Solvents were prepared by mixing and heating (~70–80 °C) the weighted components in a sealed flask until homogeneous fluids were obtained in a time-frame spanning from 10 min to 3 h [29]. The water dilutions were prepared by adding the specific weighted amounts of water to the DESs and then leaving them under magnetic stirring at 25 °C overnight in order to generate homogenous fluids [38]. The water content of the starting mixtures was measured with a Karl Fischer titrator (Metrohm 684 KF Coulometer) and the values were found to span from 0.1 to 5% *w*/*w* in the different DESs: Ethylene Glycol/Choline Chloride (EG/ChCl, 2/1 molar ratio) 1.9% *w*/*w*; Glycerol/Choline Chloride (GLY/ChCl, 2/1 molar ratio) 3.1% *w*/*w*; Urea/Choline Chloride (U/ChCl, 2/1 molar ratio) 1.6% *w*/*w*; Glycerol/Trimethylglycine (Gly/TMG, 3/1 molar ratio) 3.6% *w*/*w*; Glycolic Acid/Trimethylglycine (GA/TMG, 2/1 molar ratio) 1.9% *w*/*w*; Glycolic Acid/L-Proline (GA/L-Pro, 3/1 molar ratio) 2.1% *w*/*w*; Glycolic Acid/Choline Chloride (GA/ChCl, 2/1 molar ratio) 2.4% *w*/*w*; *p*-toluenesulfonic acid/benzyltrimethylammonium methanesulfonate (pTSA/BZA, 1/1 molar ratio) 4.6% *w*/*w*; Thymol/Decanoic Acid (THY/DEC, 2/1 molar ratio) 0.5% *w*/*w*; Phenylacetic Acid/Trimethylglycine (PhAA/TMG, 2/1 molar ratio) 1.6% *w*/*w*; Thymol/Trimethylglycine (THY/TMG, 3/1 molar ratio) 0.4% *w*/*w*; Phenylacetic Acid/N,N-dimethyl-N,N-didodecylammonium chloride (PhAA/DDDACl, 2/1 molar ratio) 2.2% *w*/*w*.

### 2.3. Heating-Ultrasound Assisted Extraction Procedure

The onion skin leaves were weighted in a vial then the DES was weighted in the same recipient. The samples were put in an oil bath at the proper temperature under magnetic stirring at 300 rpm. The sonication procedure was made by putting the samples in the ultrasound bath at room temperature (20–25 °C) for the established (evaluated) time period. Samples were then centrifugated at 25 °C for 30 min at 7000 rpm. A total 50 μL of the orange/red supernatant (Gilson P-100 pipette) was dissolved in 2 mL of ethanol in quartz cuvette for the UV-VIS analysis in the 190 nm to 1100 nm wavelength range; spectra were normalized with Microsoft Excel software. Then, 100 μL of the same supernatant was dissolved in 2 mL of ethanol for HPLC and LC-MS/MS analysis. All the samples were analyzed in triplicate and the errors evaluated via standard deviation of the three samples.

### 2.4. Onion Skin Samples

Dry onion skins from the “Dorata di Parma” cultivar were bought in a local market and processed without any further pre-treatments. The onion skin samples were desiccated under vacuum using a KNF Laboport solid PTFE vacuum pump at room temperature (20–25 °C) away from sunlight, in times spanning from 1 to 6 h until constant weight. The samples were always kept in closed containers away from light and sunlight.

In order to ensure the proper comparison of the obtained results, the sets of experiments were performed with the same batches of finely chopped onion skin (about 1 mm^2^).

### 2.5. RP-HPLC-UV Analysis

The extraction efficiencies afforded by each of the DES mixtures were evaluated through HPLC-UV-Vis analysis, by relying upon a gradient program slightly modified from a previously developed and optimized method [59]. The final gradient program was obtained from eluent A (0.1% (*v*/*v*) FA in water) and eluent B (0.1% (*v*/*v*) FA in ACN) as follows: 0 min 100% A, 0–5 min from 100% up to 97% A, 5–45 min from 97% up to 50% A, 45–50 min 0% A. At the end of each run, a column cleaning of 10 min with 100% B was added before column re-equilibration with 100% A.

### 2.6. UHPLC-MS/MS

The UHPLC analyses were performed under gradient conditions. Eluent A was water containing 0.1% (*v*/*v*) FA and eluent B methanol containing 0.1% (*v*/*v*) FA. The gradient was as follows: 0 min 1% B, 5 min 3% B, 45 min 50% B, 53 min linear gradient 100% B, and a post run time of 3 min to return to initial condition and re-equilibrate the system. The flow rate was 0.4 mL min^−1^, the injection volume was 5 μL and the column temperature was 40 °C. UV-DAD spectrum range was included between 190 and 630 nm.

The acquisition was performed in positive mode. The Dual AJS ESI gas temperature was set at 350 °C. The sheath gas temperature at 400 °C, the gas flow and the sheath gas flow at 9 mL min^−1^, the nebulizer at 35 psig, the capillary voltage at 4000 V, the nozzle voltage 0 V, the fragmentor at 120 V, the skimmer at 65 V and the Octopole RF Vpp at 750 V.

### 2.7. Quercetin Recovery

The recovery of the raw materials extracted was performed applying two different methods. With the anti-solvent procedure at the end of the centrifugation the material was filtered in folded paper filter and then diluted with water (or with 10% HCl—by volume—water solution in case of acidic dilutions for the hydrolysis of glycosylated quercetin) in a way to obtain 75% *w*/*w* water solution of the DES. Samples were left stirring (350 rpm) at room temperature (20–25 °C) overnight in order to permit the DES de-structuration and the HBD-HBA bond cleavage. The solutions were then centrifuged, the pellets collected and dried under vacuum with P_2_O_5_ and the supernatant filtered in weighted Sartorius 0.2 μm filters. The reversed-phase chromatography recover procedure was performed on the 75% *w*/*w* water (or 10% *v*/*v* HCl) solutions in Supelco Supelclean LC-18 SPE Tubes with water (3 mL) followed by methanol (3 mL).

Both the procedures were performed starting from 0.2 g of onion skin. The quercetin amounts in these crudes were then determined via HPLC analyses.

## 3. Results and Discussion

### 3.1. Optimal Deep Eutectic Solvent Design for the Quercetin Extraction

The first step of this work was the choice of the optimal solvent for the extraction of quercetin from the onion skin waste. The extraction was performed by a heating/stirring- and ultrasound-assisted procedure. In order to investigate the effectiveness of the different solvents we have firstly set-up experimental conditions commonly reported in literature [63]: heating at 50 °C and stirring at 300 rpm for 30 min then 45 min of sonication in bath followed by centrifugation of the extracts for 30 min at 7000 rpm. Afterwards, the extraction conditions were optimized as well. Because of the large number of samples to be analyzed, an UV-Vis spectra analysis of the extracts at the same dilutions was performed in turn facilitating the rapid evaluation of the extraction efficiencies of the different liquids. Absorbance values were recorded at 300 nm, which is the wavelength used to monitor the presence of molecules such as protocatechuic acid, and 366 nm, which is the typical wavelength used to study flavonoids such as quercetin and their glycosylated forms.

Differently structured DESs were used in this set of experiments, starting from commonly used ones (i.e., Urea/Choline Chloride or Glycerol/Choline Chloride mixtures and so on) [64,65] moving to other differently structured acid-based DESs (such as i.e., Glycolic Acid/Betaine, pTSA-based or Glycolic acid/L-Proline mixtures) [26,30,66].

We chose these liquids aimed at investigating the effect by neutral forms (glycerol- or glycol-based) as well as slightly acidic (Glycolic Acid-based) and highly acidic ones (*p*-toluensulphonic acid based). This heterogeneous selection intended to appraise whether a form was more capable than another to favor the hydrolysis of the glycosylated forms of the extracted flavonoids. Moreover, hydrophobic DESs mixtures were tested to evaluate the water solubility effect of the liquids on the extraction efficacy, considering the low solubility of the quercetin itself in water [18,67].

**Figure 1:**
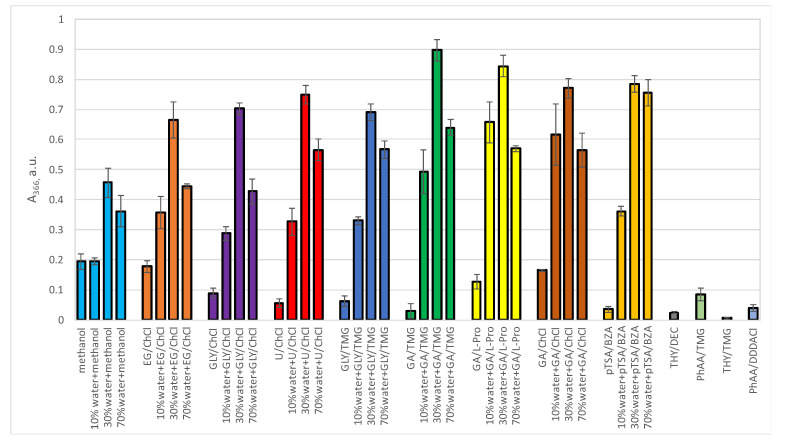
UV-Vis Absorbance at λ = 366 nm of the supernatants of the extraction of onion skin diluted in ethanol (50 μL in 2 mL EtOH). Extraction conditions: 50 mg of onion skin in 1.5 g of aqueous DES, heating and stirring (50 °C, 300 rpm) for 30 min then 45 min of sonication in bath followed by centrifugation of the extracts for 30 min at 7000 rpm. EG/ChCl 2/1 molar ratio; GLY/ChCl 2/1 molar ratio; U/ChCl 2/1 molar ratio; Gly/TMG 3/1 molar ratio; GA/TMG 2/1 molar ratio; GA/L-Pro 3/1 molar ratio; GA/ChCl 2/1 molar ratio; pTSA/BZA 1/1 molar ratio; THY/DEC 2/1 molar ratio; PhAA/TMG 2/1 molar ratio; THY/TMG 3/1 molar ratio; PhAA/DDDACl 2/1 molar ratio. Water amounts are considered as added water to the starting DESs (initial water amounts spanning from 0.1% to 5% *w*/*w*).

The liquids tested were: Ethylene Glycol/Choline Chloride (EG/ChCl, 2/1 molar ratio); Glycerol/Choline Chloride (GLY/ChCl, 2/1 molar ratio); Urea/Choline Chloride (U/ChCl, 2/1 molar ratio); Glycerol/Trimethylglycine (Gly/TMG, 3/1 molar ratio); Glycolic Acid/Trimethylglycine (GA/TMG, 2/1 molar ratio); Glycolic Acid/L-Proline (GA/L-Pro, 3/1 molar ratio); Glycolic Acid/Choline Chloride (GA/ChCl, 2/1 molar ratio); *p*-toluenesulfonic acid/benzyltrimethylammonium methanesulfonate (pTSA/BZA, 1/1 molar ratio); Thymol/Decanoic Acid (THY/DEC, 2/1 molar ratio); Phenylacetic Acid/Trimethylglycine (PhAA/TMG, 2/1 molar ratio); Thymol/Trimethylglycine (THY/TMG, 3/1 molar ratio); Phenylacetic Acid/N,N-dimethyl-N,N-didodecylammonium chloride (PhAA/DDDACl, 2/1 molar ratio). In addition to the above mixtures, neat methanol was also used as extraction solvent for comparative purposes. Indeed, this alcohol is commonly used for the polyphenol extraction from natural sources [68,69]. In order to evaluate the advantages of the aqueous solutions of DESs (such as the fine tuning of the overall viscosity and the polarity extent), water additions were also tested. DESs undergo structural changes by adding water in amounts that are dependent on the structural features of the components and on their interactions. Thus, three different dilutions were scrutinized using three different amounts of water (10%, 30% and 70% *w*/*w*) in each solvent; considering that with starting water amounts from 0.1% to 5% in the liquids, with these values it is possible to easily stay between the values of water that determine structural changes in the DESs solutions [38,70,71,72]. The hydrophobic DESs were tested as such, without any water addition: indeed, they generally do not absorb water contents higher than 10% *w*/*w* [18]. Water dilutions of methanol were also tested. In Figure 1, the results of absorbance of the samples at 366 nm are reported, while the absorbance values at 300 nm of the same samples are reported in the Supplementary Materials (Figure S1) as well as UV-Vis spectra of a typical sample and of all the samples (Figure S2).

From the UV-Vis analyses on the raw extracted material, it is evident that all the pure non-diluted DESs have extraction efficacies lower than methanol. EG/ChCl DES showed the highest extraction efficacy in its pure non-diluted form compared to the other pure liquids. However, these values increase steeply by addition of water: the absorbance values were more than doubled with 10% *w*/*w* water, with the highest values recorded at 30% *w*/*w*; then, a decrease did occur with 70% *w*/*w* of added water. This trend is coherent with the structural properties of the water dilutions of DESs as the lowering of the liquid viscosity, which follows the increasing amounts of water promoting an easier mass transfer (that is, the extraction power) from the onion skin. This is also suggested by the low solubility of quercetin (or similar phenols contained in the onion skin) in water, that therefore could be easily extracted from onion skin thanks to the lower viscosity and then it could be solubilized in the DESs domains. The HBD-HBA bond cleavage and following DESs structures disruptions at values of 70% *w*/*w* of added water led to a decrease on the extraction efficiency, even though it remained higher than that produced both by neat methanol and water-methanol solutions. The negative inflection of the extraction trend could be due to interactions of the phenols with the DESs isolated components. The changes in the polarity of the media did not play a significant role as the trend of the A_366_ of the water dilutions was the same for all the differently structured polarities. The water additions, in fact, lead to changes in polarity that lead progressively to the polarity of water itself by increasing its amount [38]. In this case some of the solvents could have higher polarity than water and other lower ones, so the trends could have been different. The hydrophobic DESs (THY/DEC, PhAA/TMG, THY/TMG, PhAA/DDDACl) showed lower extraction efficacies as they could not benefit from the advantaged resulting from the water addition.

The acidity of the HBD in the DESs plays a role in their extraction efficiency as the common non-acid liquids (EG/ChCl, GLY/ChCl, U/ChCl, Gly/TMG) showed absorbances at 366 nm lower than the ones with acidic HBDs such as glycolic acid-based liquids (GA/TMG, GA/L-Pro, GA/ChCl) and the pTSA-based one (pTSA/BZA). HBA seems to play a less relevant role as the most important differences were observed by changing the HBD.

The best extracting liquid in our set was found to be the glycolic acid/betaine (GA/TMG) mixture, already known in the literature for its multiple advantageous uses [73].

The absorbances at 300 nm were higher than those at 366 nm, but the A_366_/A_300_ trend was identical for all the samples, therefore suggesting the non-selective extraction of the different DESs in the set.

From the data reported in Figure 1 it emerges that all the DESs in their water dilutions at 30% (*w*/*w*) are much more efficient than methanol. In particular GA/TMG showed more than 4.5 times higher extraction efficiency of the raw material compared with methanol and over twice compared with methanol with 30% (*w*/*w*) water. These data strongly promote the use of the water dilutions of DESs as an efficacious and green method for the extraction of important phenolic compounds from dry onion skin. In Figure S3 in Supplementary Materials, the ratio of A_366_ of the samples on the A_366_ of pure methanol and of methanol with 30% (*w*/*w*) of added water are reported in order to evaluate the efficacy of the DESs water dilutions compared to methanol (the same data at 300 nm is reported in the same figure).

### 3.2. Extraction Conditions Optimization

**Figure 2:**
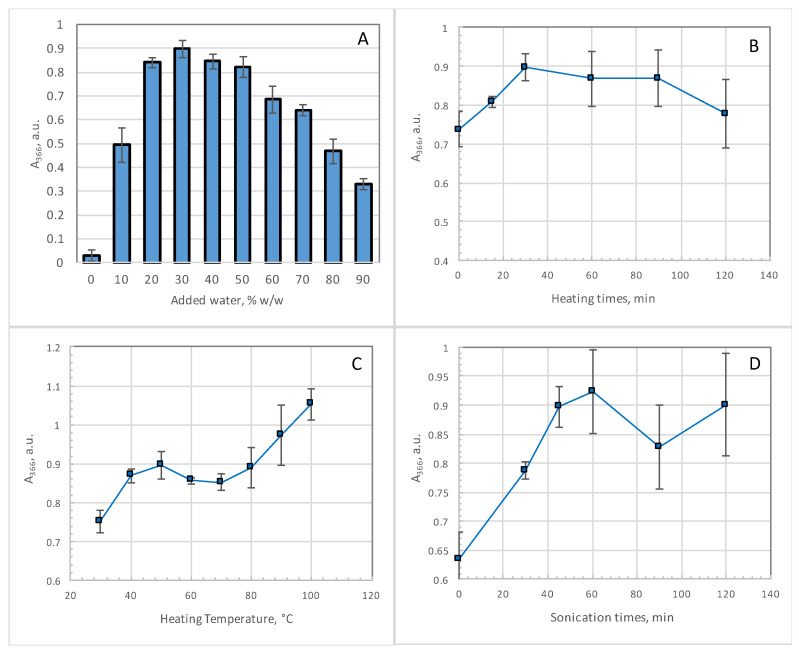
Optimization of the extraction procedure, GA/TMG DES + 30% *w*/*w* added water, 50 mg of onion skin in 1.5 g of DES. (**A**): optimization of the water amount on GA/TMG DES, heating and stirring (50 °C, 300 rpm) for 30 min then 45 min of sonication in bath followed by centrifugation of the extracts for 30 min at 7000 rpm. (**B**): optimization of heating times, heating and stirring (50 °C, 300 rpm) then 45 min of sonication in bath followed by centrifugation of the extracts for 30 min at 7000 rpm. (**C**): optimization of heating temperature, heating and stirring (300 rpm) for 30 min then 45 min of sonication in bath followed by centrifugation of the extracts for 30 min at 7000 rpm. (**D**): optimization of sonication times, heating and stirring (50 °C, 300 rpm) for 30 min then sonication in bath followed by centrifugation of the extracts for 30 min at 7000 rpm. All the measures are averages of triplicates and the error bars are standard deviations of the set.

Once the optimal DESs were found, the optimization of the extraction conditions was made by changing (i) the amount of water added to the DESs extracting liquids (this time, a more extended water content was evaluated), (ii) the time of heating, (iii) the temperature of heating and (iv) the sonication time. All these procedures were performed sequentially, according to the one-variable-at-time (OVAT) approach. These experiments were performed with the system GA/TMG as this DES emerged as best performing one for the scope of the present work. Moreover, it is also characterized by a low cost and easy preparation. In these experiments, the estimation of the extraction efficacy from the dry onion skin was also evaluated via UV-Vis analysis at 366 nm. The UV-Vis absorbances at 300 nm gave the same trends and therefore these values are not reported herein. In Figure 2, the results of the optimization steps are reported; the optimization of the ratio of the mass of onion skin on the mass of the extracting DESs was also performed, but it did not show any relevant trend (see Supplementary Materials, Figure S4). Therefore, the following amounts were maintained also in this part of the study: 50 mg of onion skin with 1.5 g of DES.

**Figure 3:**
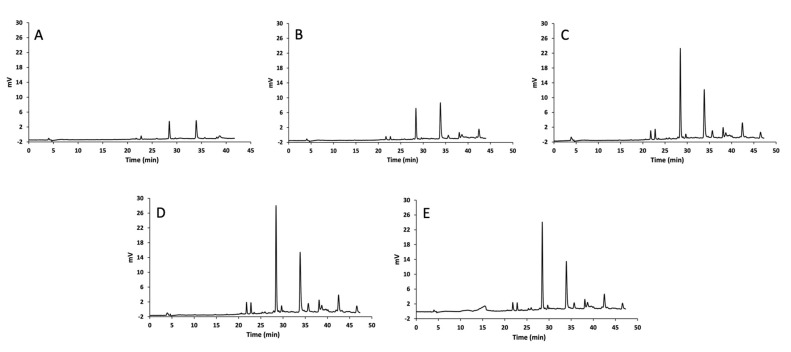
Chromatograms of the HPLC-UV-Vis analysis on onion extracts at 360 nm. (**A**): pure MeOH, (**B**): MeOH 30% *w*/*w* water, (**C**): GA/TMG +30% *w*/*w* water, (**D**): GA/L-Pro + 30% *w*/*w* water, (**E**): pTSA/BZA + 30% *w*/*w* water.

The first parameter considered was the amount of water added to the DESs (Figure 3A); in this framework, a set of ten experiments was performed from 0% to 90% (*w*/*w*) added water. The best result obtained in terms of absorbance at 366 nm was at 30% *w*/*w*, the same value that turned out in the previous step dealing with the screening of the various DESs. With this water amount the heating time at 50 °C was then evaluated in a time-frame of 120 min (Figure 3B). The best result was again that previously identified during the screening stage, that is, 30 min. Then, the heating temperature was varied in the range 25 °C–100 °C (Figure 3C). In this case, an almost constant value of A_366_ was observed from 50 °C to 80 °C, then an increase was recorded corresponding to a browning of the extracting solution and difficult operating conditions (impossible separation of the onion matrix after centrifugation). Therefore, the temperature was set as optimal at 50 °C because it is the lowest temperature that gave the optimal results without browning of the solutions. The only parameter that was changed from the starting conditions was the sonication time because it showed an increase of A_366_ without any experimental drawback (Figure 3D): an increase from 45 min to 1 h showed an increase of the extracted raw material. Therefore, the optimal extraction conditions were: 50 mg of onion skin in 1.5 g of 30% *w*/*w* of added water in DESs, heating and stirring at 50 °C for 30 min, followed by sonication of 1 h and then centrifugation of the sample for 30 min at 7000 rpm.

The samples of GA/TMG, GA/L-Pro and pTSA/BZA, obtained by applying these experimental conditions, were then submitted to HPLC analysis (see Section 3.3 for details). Methanol and its water dilutions were also considered for comparative purposes.

### 3.3. RP-HPLC-UV Analysis

In order to characterize the qualitative and quantitative profile of each onion extract, a HPLC-UV (wavelength of detection 360 nm) analysis was firstly carried out followed by an UHPLC-MS investigation for the identification of the main peaks.

The chromatographic profiles clearly evidenced the presence of two main peaks (Figure 3). The first one, with a retention time of about 29 min, was plausibly ascribed to a glycosylated form of quercetin and successively confirmed and characterized by UHPLC-MS analysis (see Section 3.3.1 for details). In fact, besides the aglycone of quercetin, diglucosides (mainly quercetin-3,4′-*O*-diglucoside) and glucosides derivatives (mainly quercetin-4′-*O*-diglucoside) represent the predominant forms in different onion varieties [74,75,76]. Moreover, the prevalence of the mono-glycosilated quercetin with respect to the di-glycosylated form could reasonably originate from a hydrolytic cleavage occurring during the extraction. The second main peak was identified as the quercetin aglycone, based on the correspondence of peak retention time (around 34 min) with that of the reference standard.

The applied gradient program produced a profitable separation of the selected peaks from other minor compounds or matrix interferences. This in turn allowed the reliable quantitation of quercetin, its extraction being the focus of the present study. A noteworthy major content of quercetin was always recovered with the use of different DESs mixtures if compared to more conventional extraction protocols operated with pure methanol or its hydro-alcoholic mixtures.

The exemplary chromatograms of onion skin extracts submitted to conventional methods or DES-based extraction protocols are shown in Figure 3. The results also evidenced a higher content of glycosylated quercetin provided by DESs extractants, thus underlying their effectiveness and selectivity towards such class of flavonoids with respect to traditional methods.

#### 3.3.1. UHPLC-MS/MS

**Figure 4:**
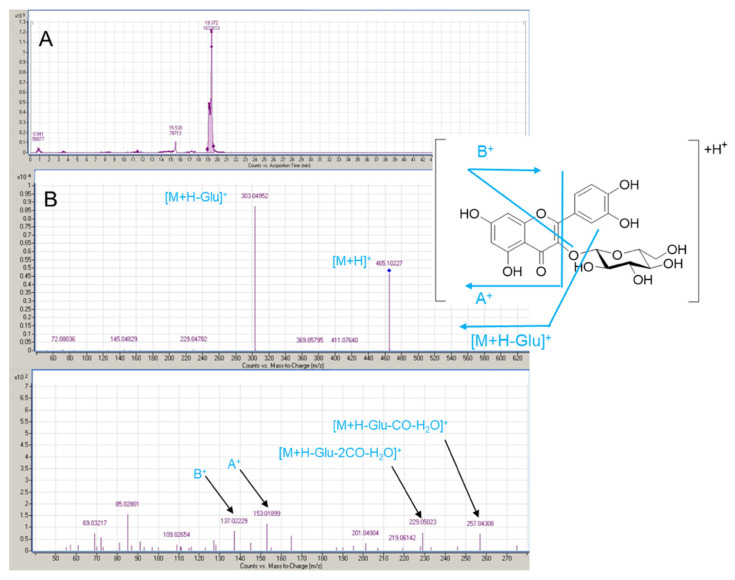
LC-MS/MS analysis of the peak at lower retention time of GLY/L-Pro + 30% added water sample. (**A**): LC chromatogram; (**B**): MS and MS/MS fragmentations of the peak at 19 min retention time.

According to HPLC results, the UHPLC-MS/MS analysis was focused on the identification of peak at lower retention time and with UV absorption at 366 nm. The sample analyzed was the one extracted with GA/L-Pro + 30% added water liquid. The results are reported in Figure 4.

The MS spectra show a peak with *m*/*z* value of 465.1029 that corresponds to pseudomolecular ion [M+H]^+^ of a compound with chemical formula C_21_H_20_O_12_. The [M+H]^+^ fragmentation pattern is consistent with the loss of a glycosyl or galactosyl due to the presence of the fragment with *m*/*z* 303.0493. The presence of fragments with *m*/*z* at 257.0430, 229.0502, 153.0189, 137.0223 are related to the fragmentation of quercetin moiety as reported in literature [77,78] and they confirm that the compound is a glycosylated form of quercetin.

#### 3.3.2. Quantitation of Quercetin in the Investigated Extracts

The quantitation of quercetin in all the investigated extracts was performed by relying upon a calibration curve built up by using standard solutions with concentration values spanning in the range specified in Table S1 (Supplementary Materials). As evident by the R^2^ value, the obtained mathematical models were characterized by a very good linearity. The established HPLC method was further validated in terms of accuracy, precision, and limit of detection (LOD) and limit of quantification (LOQ) (Tables S1 and S2, Supplementary Materials). Accordingly, high recovery% values (from 98.15% up to 99.56%) and low range of variation of the RSD% values (from 0.83% up to 1.04%) were observed when the long-term (inter-day) accuracy and precision were evaluated, respectively (Table S2, Supplementary Materials). Additionally, appreciably low LOD (0.37 µg/mL) and LOQ (1.11 µg/mL) values were calculated for quercetin samples (Table S1, Supplementary Materials). The obtained results are comparable with data reported in literature [79,80]. For example, the validation data of the HPLC method for quercetin determination in green tea reported by Savic and co-workers showed accuracy values between 98.2 and 101.3%, RSD% values in the range 0.89–1.55%, and LOD and LOQ values of 1.2 and 4.0 μg/mL, respectively [81].

The consistent and reliable outcomes achieved with the validation process revealed the adequacy of the analytical method to be applied for quantitative purposes.

The results shown in Table 1 highlight a certain selectivity in terms of extracted compounds by DESs characterized by a higher content of quercetin in all the extracts with respect to the traditional media. In particular, the mixture GA/L-Pro produced the highest quercetin recovery. Interestingly, it is worth noting that the ratio between the glycosylated form and the aglycone one was kept quite constant when extractions were performed with DESs systems. In these cases, the ratio between the two forms was around 1.4 with a slight prevalence of the glycosylated form over the aglycone one. On the contrary, despite the inverted ratio found when pure methanol or a methanol/water mixture was employed, a lower total recovery was reached.

### 3.4. Quercetin Recovery

The recovery of the quercetin from the extraction matrix was performed with two different methodologies: water anti-solvent method and SPE (solid phase extraction) method. In both protocols it was considered that at water amounts over 75% (*w*/*w*) the DESs structures are disrupted and the bonds HBA-HBD are cleaved; in these conditions the limited water solubility of quercetin can be exploited for its separation from the hydrophilic media. Therefore, the experiments were carried out in the optimized conditions and then water was added in order to get to 75% (*w*/*w*) of added water. Then, the samples were left stirring at room temperature (20–25 °C) overnight and finally treated in the two different methodologies. With anti-solvent technique, the samples were centrifuged and the solid phases separated, the supernatants filtered in weighted 0.2 μm filters and the solid phases diluted in the proper amounts of ethanol to perform the HPLC quantitative analyses. The SPE was performed via loading of the sample in the reversed-phase cartridge and then the products were recovered with methanol (3 mL) wash. The same experiments were conducted with HCl 10% (*w*/*w*) water solution instead of water; in this way an increase of the non-glycosylated form could be collected because of acid hydrolysis. The anti-solvent procedure gave 0.16 g of raw material starting from 0.2 g of onion leaves and the reverse-phase chromatography gave 0.016 g of raw material starting from 0.2 g of onion skin.

The yields of quercetin recovery, calculated as amount of quercetin obtained on the amount of quercetin extracted both evaluated via HPLC analyses, are reported in Table 2. In the same table, the data coming from the experiments conducted with HCl 10% (*w*/*w*) are reported in terms of ratio glycosylated/non-glycosylated forms. This is because in these cases it is not possible to calculate a yield because of the increasing amount of non-glycosylated quercetin.

The SPE (solid phase extraction) method gave excellent yield of recovery of quercetin (81%), while the water anti-solvent method showed low performances of recovery (8%). The low values observed with anti-solvent method could be due to interactions occurring between the DES components (glycolic acid and trimethylglycine) and the quercetin that could lead to more water-soluble adduct not allowing the precipitate formation after centrifugation. Polyphenols extraction procedures made by DESs are known to occur thanks to weak interactions occurring between the phenols and the network of weak forces in the DESs liquids [24,82]. This is supported by the values of A_366_ in extraction procedures observed with 70% (*w*/*w*) of added water in the optimal DES design that were in fact still over the methanol extraction even if lower than the maximum observed at 30% (*w*/*w*) added water.

In the SPE method, excellent values of recovery were observed (81%) but methanol was used for the recovery after the wash in the SPE cartridge. However, the amounts of extracted material with the DESs water dilutions showed values that are over 4.5 and 2 times higher than the ones of methanol or methanol with 30% (*w*/*w*) of added water when used as extracting agents. If the recovery efficacy is normalized on the amounts of methanol used (1.89 mL in case of extraction with methanol and 3 mL in case of DES) the procedure is still advantageous because the extraction efficacy is 4.5 times higher with almost twice the methanol used, therefore it is almost three times more efficacious. Moreover, when used as extracting liquid, methanol is heated to temperatures close to its boiling point, therefore implying peculiar attention to the experimental conditions (aspirating hoods, flammability of the media, toxicity of the vapors and so on) that increase in the case of industrial scale-up of the process. This feature undoubtedly promotes the DES-water system for the inherent extraction efficiency and overall greenness of the method.

The use of HCl 10% (*w*/*w*) water solutions instead of the simple water for the dilutions, led to an increase of the amount of non-glycosylated form as expected; in this case, the yields of recovery was not calculated as the amounts of quercetin recovered were also higher than the ones initially extracted.

## 4. Conclusions

In this work water dilutions of a set of Deep Eutectic Solvents (DESs) revealed to be excellent and efficacious green media for the selective extraction of quercetin and its glycosylated form from onion skin, a low-cost waste material. As reported, the best results were obtained with the use of acidic components in the DESs liquids (GA/TMG, GA/L-Pro and pTSA/BZA). Glycolic acid-based ones can be considered NADESs, therefore their greenness is increased over the pTSA-based one that moreover has strong acidity in its components. However, no effect on the ratio aglycone/glycosylated forms was observed by changing the acidic strength in the liquids even though the O-glycosidic bond can usually be hydrolyzed in acidic conditions, therefore suggesting a different mechanism of extraction. The procedure revealed to be much more effective than the use of methanol, a highly toxic and volatile solvent commonly used in the extraction of polyphenols from vegetal matrixes. The quercetin concentration in the samples (in the aglycone form only) were in fact over three times higher than methanol as emerged from HPLC analyses (5.84 µg/mL with methanol compared to 18.56 µg/mL with GA/L-Pro and over 14 µg/mL for GA/TMG and pTSA/BZA samples) and more than 1.5 times higher using the water/methanol mixture (10.83 µg/mL). The extracted materials were also recovered efficaciously with solid phase extraction method with excellent yields (81%) of recovery.

The proposed extraction protocol revealed to be green, efficacious and selective for the extraction from onion skin of quercetin, a molecule that is gaining importance for properties such as its pharmacological activity.

## Figures and Tables

**Table 1:** Quantitation of quercetin amounts and glycosylated/aglycone quercetin ratio in the analyzed samples.

Onion Extract	Quercetin Mean Conc. ± SD (µg/mL)	Glycosylated/Aglycone—Quercetin Ratio
MeOH	5.84 ± 0.13	43/57
MeOH + 30% *w*/*w* water	10.83 ± 0.01	40/60
GA/TMG + 30% *w*/*w* water	14.79 ± 0.50	58/42
GA/L-Pro + 30% *w*/*w* water	18.56 ± 0.25	58/42
pTSA/BZA + 30% *w*/*w* water	14.83 ± 0.31	59/41

**Table 2:** Recovery of quercetin from the extracted samples evaluation via HPLC analyses.

Recovery Procedure	Sample	Quercetin Mean Conc. ± SD (µg/mL)	Yield of Recovery, %	Glycosylated/Aglycone—Quercetin Ratio H_2_O Recover	Glycosylated/Aglycone—Quercetin Ratio HCl 10% *w*/*w* Recover
SPE	extracted	12.83 ± 0.01 ^a^	81%	52/48	40/60
	recovered	11.68 ± 0.39 ^b^			
Anti-Solvent	extracted	13.88 ± 0.02 ^c^	8%	38/62	29/71
	recovered	4.49 ± 0.01 ^d^			

Extracting liquid GA/TMG + 30% *w*/*w* added water (density 1.1941 g/mL), heating and stirring at 50 °C for 30 min, 1-h sonication, centrifugation of the sample for 30 min at 7000 rpm, filtration of the sample with water amounts to give 75% *w*/*w* of added water left stirring overnight. Yields of recovery calculated as percent of recover from the extracted material reported at the same dilutions. Dilutions made in order to give values of areas of HPLC analyses inside the calibration curve. Glycosylated/Aglycone—Quercetin Ratio calculated as ratio of HPLC peaks area in the recover procedure with water (H_2_O recover) or with 10% *w*/*w* HCl in water solution (HCl 10% *w*/*w* recover column). ^a^ = 50 µL of sample from 1.2587 mL extracting DES batch dissolved in 2 mL of EtOH; ^b^ = 140 µL of sample from a total 2 mL EtOH batch dissolved in 2 mL of EtOH; ^c^ = 50 µL of sample from 5.3641 mL extracting DES batch dissolved in 2 mL of EtOH; ^d^ = 200 µL of sample from a total 5 mL EtOH batch dissolved in 2 mL of EtOH.

## Data Availability

The data presented in this study are available on request from the corresponding author.

## Supplementary Materials

The following are available online at https://www.mdpi.com/article/10.3390/ma14216465/s1, Figure S1: UV-Vis Absorbance at λ = 300 nm of the supernatants of the extraction of onion skin. Figure S2: UV-Vis spectra of MeOH, MeOH +30% added water and GA/TMG + 30% added water samples—UV-Vis spectra of all the extraction samples. Figure S3: ratio of UV-Vis A_300_ nm and A_366_ nm sample/methanol and sample/methanol + 30% *w*/*w* water added. Figure S4: ratio of the mass of onion skin on the mass of the extracting DESs optimization. Table S1: HPLC Calibration data. Table S2: HPLC Method validation.
