# Supplementary material for: Effective and Selective Extraction of Quercetin from Onion (Allium cepa L.) Skin Waste Using Water Dilutions of Acid-Based Deep Eutectic Solvents
Source: Materials (Basel). 2021 Oct 28;14(21):6465. doi: 10.3390/ma14216465 (PMC8585411; doi:10.3390/ma14216465)
Supplement: Supplementary file 1 [file materials-14-06465-s001.zip › materials-1421271-supplementary.pdf]

materials

Supplementary Information

Effective and Selective Extraction of Quercetin from Onion (Allium cepa L.) Skin Waste Using Water Dilutions of Acid-based Deep Eutectic Solvents

Matteo Ciardi[1†], Federica Ianni[2†], Roccaldo Sardella[2,3], Stefano Di Bona[1], Lina Cossignani[2,3], Raimondo Germani[1], Matteo Tiecco[1*] and Catia Clementi[1]

[1] Department of Chemistry, Biology and Biotecnology, University of Perugia, Via Elce di Sotto 8, 06123 Perugia, Italy; matteo.ciardi95@gmail.com (M.C.); dibonastefano@gmail.com (S.D.B.); raimondo.germani@unipg.it (R.G.); catia.clementi@unipg.it (C.C.)
[2] Department of Pharmaceutical Sciences, University of Perugia, Via Fabretti 48, 06123 Perugia, Italy; federica.ianni@unipg.it (F.I.); roccaldo.sardella@unipg.it (R.S.); lina.cossignani@unipg.it (L.C.)
[3] Center for Perinatal and Reproductive Medicine, University of Perugia, Santa Maria della Misericordia University Hospital, 06132 Perugia, Italy
* Correspondence: matteotiecco@gmail.com
† These authors contributed equally to this paper.

Figure S1. UV-Vis Absorbance at λ = 300 nm of the supernatants of the extraction of onion skin.

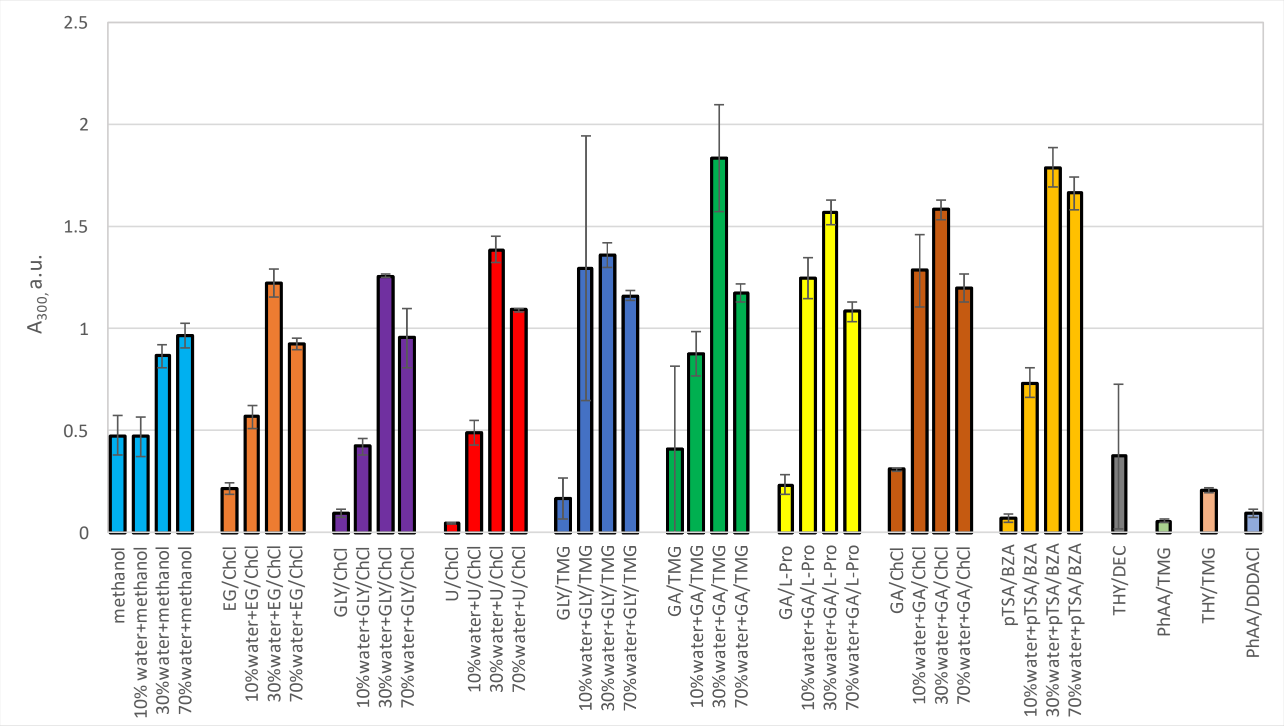

Figure S1. UV-Vis Absorbance at λ = 300 nm of the supernatants of the extraction of onion skin diluted in ethanol (50 µL in 2 mL EtOH). Extraction conditions: 50 mg of onion skin in 1.5 g of aqueous DES, heating and stirring (50°C, 300 rpm) for 30 mins then 45 mins of sonication in bath followed by centrifugation of the extracts for 30 mins at 7000 rpm. **EG/ChCl** = Ethylene Glycol/Choline Chloride (2/1 molar ratio); **GLY/ChCl** = Glycerol/Choline Chloride (2/1 molar ratio); **U/ChCl** = Urea/Choline Chloride (2/1 molar ratio); **Gly/TMG** = Glycerol/Trimethylglycine (3/1 molar ratio); **GA/TMG** = Glycolic Acid/Trimethylglycine (2/1 molar ratio); **GA/L-Pro** = Glycolic Acid/L-Proline (3/1 molar ratio); **GA/ChCl** = Glycolic Acid/Choline Chloride (2/1 molar ratio); **pTSA/BZA** = p-toluenesulfonic acid/benzyltrimethylammonium methanesulfonate (1/1 molar ratio); **THY/DEC** = Thymol/Decanoic Acid (2/1 molar ratio); **PhAA/TMG** = Phenylacetic Acid/Trimethylglycine (2/1 molar ratio); **THY/TMG** = Thymol/Trimethylglycine (3/1 molar ratio); **PhAA/DDDACl** = Phenylacetic Acid/N,N-dimethyl-N,N-didodecylammonium chloride (2/1 molar ratio). Water amounts are considered as added water to the starting DESs (initial water amounts spanning from 0.1% to 5% w/w).

Figure S2. UV-Vis spectra of the extraction samples a) **MeOH**, **MeOH** +30% added water and **GA/TMG** + 30% added water samples; b) all the extraction samples.

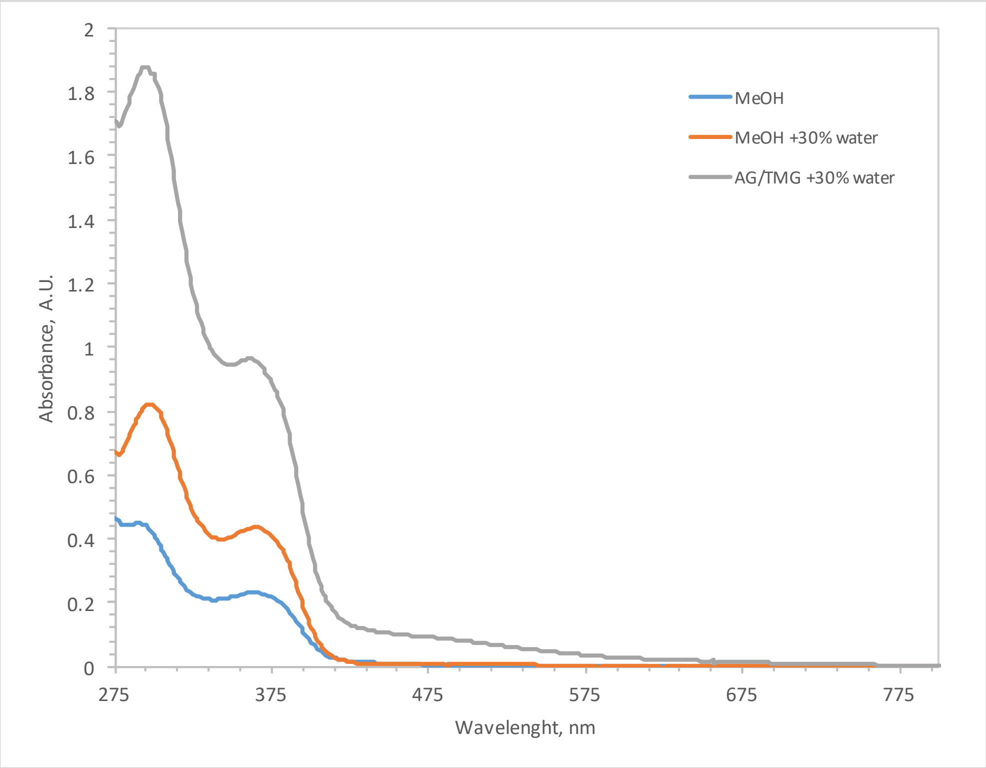

Figure S2. a: UV-Vis spectra of **MeOH**, **MeOH** +30% added water and **GA/TMG** + 30% added water samples: supernatants in ethanol (50 µL in 2 mL EtOH). Extraction conditions: 50 mg of onion skin in 1.5 g of aqueous DES, heating and stirring (50°C, 300 rpm) for 30 mins then 45 mins of sonication in bath followed by centrifugation of the extracts for 30 mins at 7000 rpm.

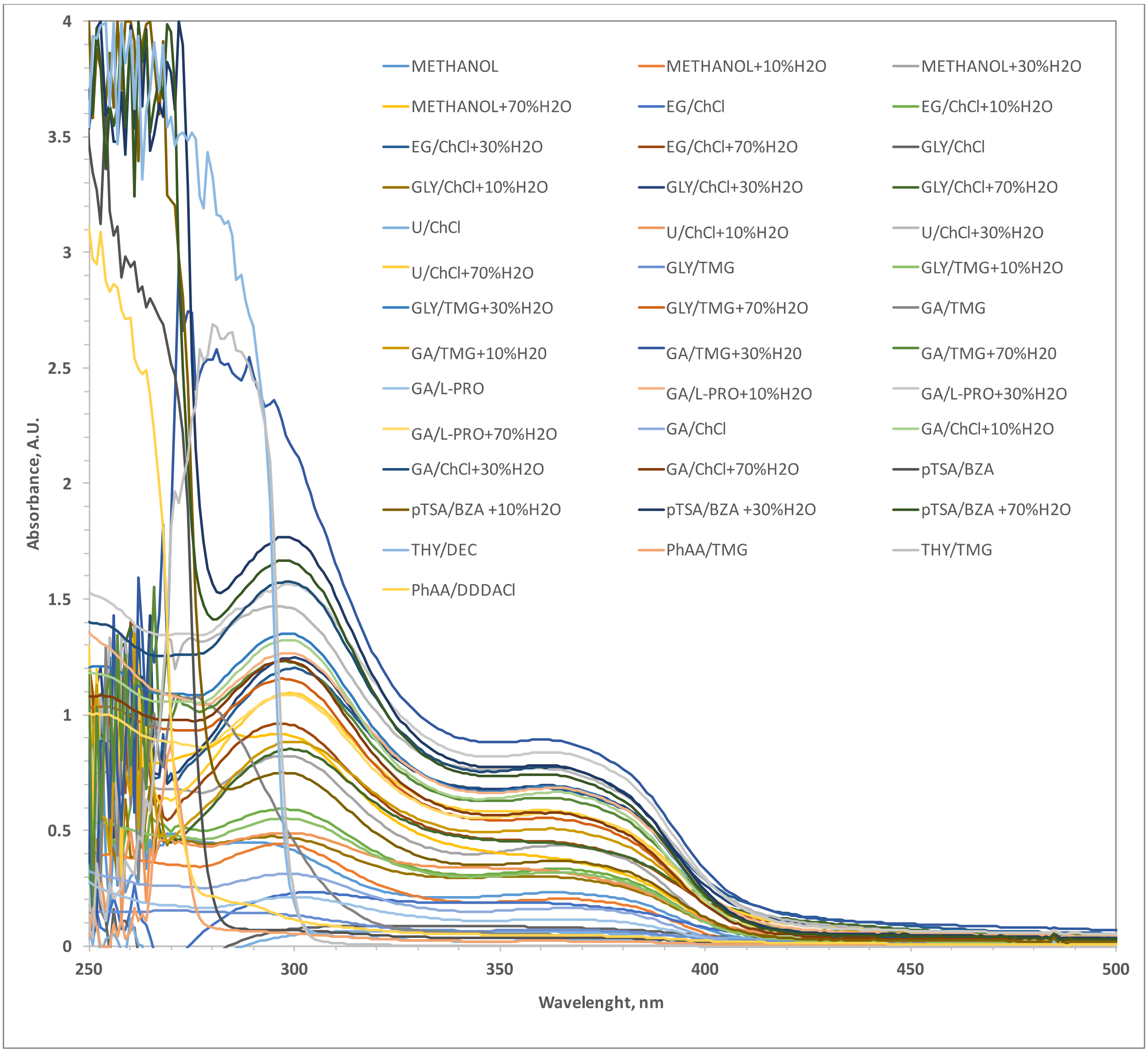

Figure S2. b: UV-Vis spectra of all the extraction samples: supernatants in ethanol (50 µL in 2 mL EtOH). Extraction conditions: 50 mg of onion skin in 1.5 g of DES (and their water dilutions), heating and stirring (50°C, 300 rpm) for 30 mins then 45 mins of sonication in bath followed by centrifugation of the extracts for 30 mins at 7000 rpm.

Figure S3: ratio of UV-Vis A366 nm and A300 nm sample/methanol and sample/ methanol + 30% w/w water added.

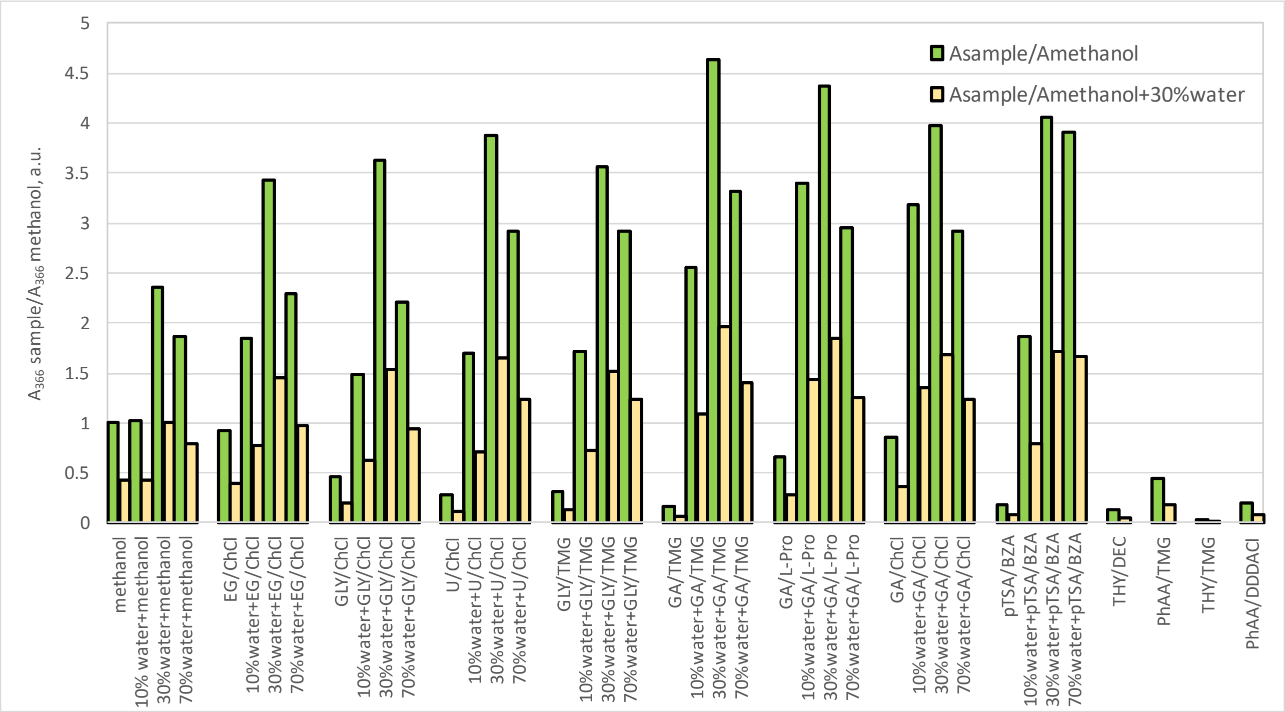

Figure S3. a. Ratio of UV-Vis A366 nm sample/methanol (and methanol + 30% w/w water added) of the supernatants of the extractions of onion skin. All the samples were diluted in ethanol (50 µL in 2 mL EtOH). Extraction conditions: 50 mg of onion skin in 1.5 g of aqueous DES, heating and stirring (50°C, 300 rpm) for 30 mins then 45 mins of sonication in bath followed by centrifugation of the extracts for 30 mins at 7000 rpm. **EG/ChCl** 2/1 molar ratio; **GLY/ChCl** 2/1 molar ratio; **U/ChCl** 2/1 molar ratio; **Gly/TMG** 3/1 molar ratio; **GA/TMG** 2/1 molar ratio; **GA/L-Pro** 3/1 molar ratio; **GA/ChCl** 2/1 molar ratio; **pTSA/BZA** 1/1 molar ratio; **THY/DEC** 2/1 molar ratio; **PhAA/TMG** 2/1 molar ratio; **THY/TMG** 3/1 molar ratio; **PhAA/DDDACl** 2/1 molar ratio. Water amounts are considered as added water to the starting DESs (initial water amounts spanning from 0.1% to 5% w/w).

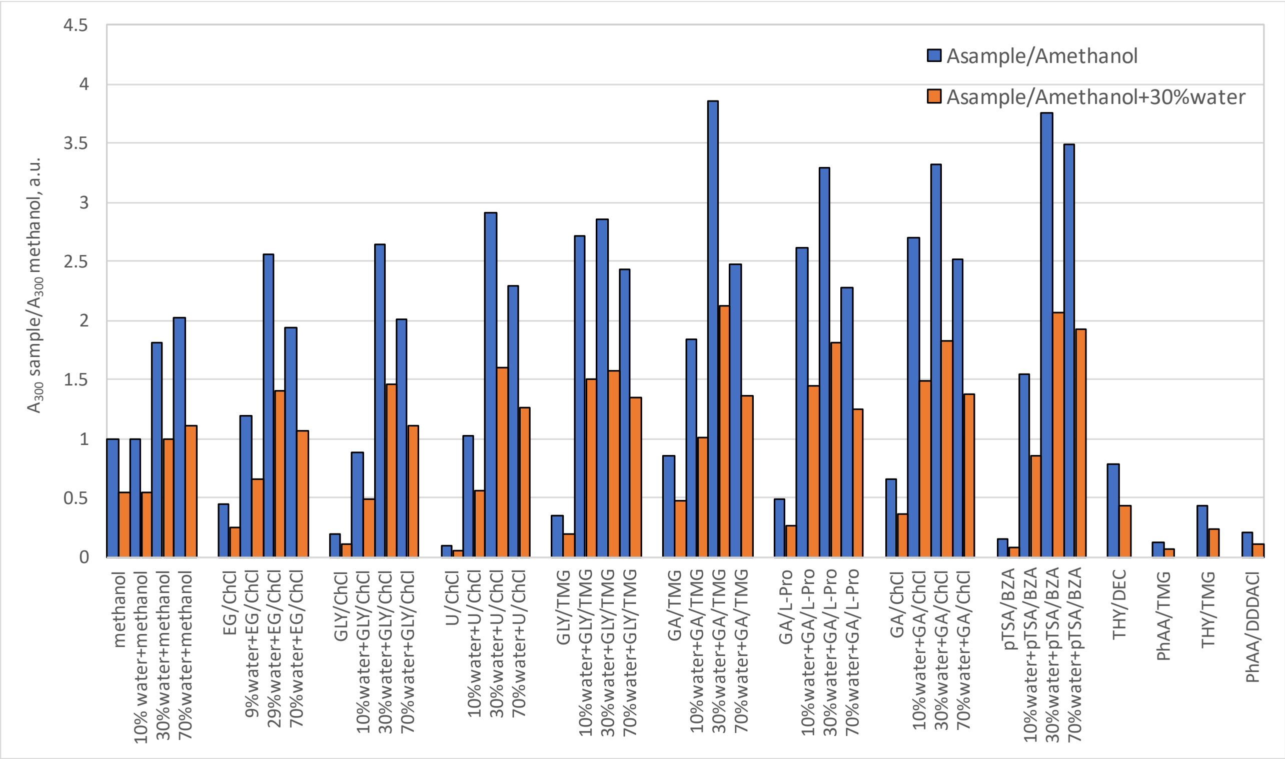

Figure S3. b: ratio of UV-Vis A_{300} nm sample/methanol (and methanol + 30% w/w water added) of the supernatants of the extractions of onion skin. All the samples were diluted in ethanol (50 µL in 2 mL EtOH). Blue line at ratio = 1 for comparison of the efficacies. Extraction conditions: 50 mg of onion skin in 1.5 g of aqueous DES, heating and stirring (50°C, 300 rpm) for 30 mins then 45 mins of sonication in bath followed by centrifugation of the extracts for 30 mins at 7000 rpm. **EG/ChCl** = Ethylene Glycol/Choline Chloride (2/1 molar ratio); **GLY/ChCl** = Glycerol/Choline Chloride (2/1 molar ratio); **U/ChCl** = Urea/Choline Chloride (2/1 molar ratio); **Gly/TMG** = Glycerol/Trimethylglycine (3/1 molar ratio); **GA/TMG** = Glycolic Acid/Trimethylglycine (2/1 molar ratio); **GA/L-Pro** = Glycolic Acid/L-Proline (3/1 molar ratio); **GA/ChCl** = Glycolic Acid/Choline Chloride (2/1 molar ratio); **pTSA/BZA** = p-toluenesulfonic acid/benzyltrimethylammonium methanesulfonate (1/1 molar ratio); **THY/DEC** = Thymol/Decanoic Acid (2/1 molar ratio); **PhAA/TMG** = Phenylacetic Acid/Trimethylglycine (2/1 molar ratio); **THY/TMG** = Thymol/Trimethylglycine (3/1 molar ratio); **PhAA/DDDACl** = Phenylacetic Acid/N,N-dimethyl-N,N-didodecylammonium chloride (2/1 molar ratio). Water amounts are considered as added water to the starting DESs (initial water amounts spanning from 0.1% to 5% w/w).

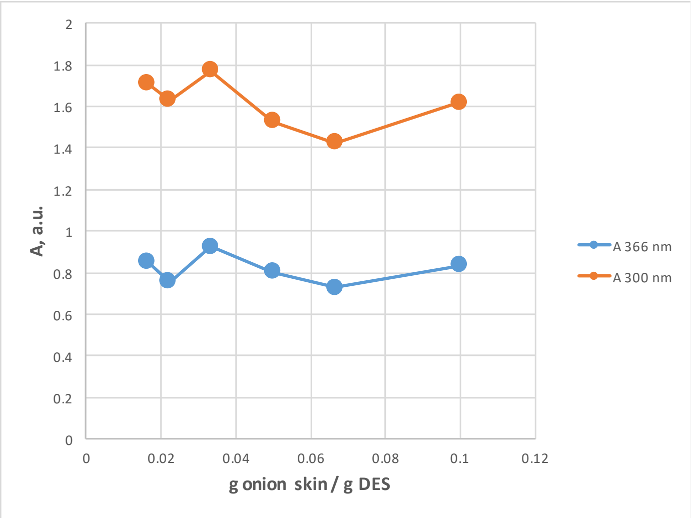

Figure S4. optimization of the ratio of onion skin on grams of extracting DESs on the extraction efficacies in terms of UV-Vis absorbance at 300 nm (orange) and at 366 nm (blue).

Table S1. Calibration data: regression equation, correlation coefficient value (R^2), linearity range, LOD and LOQ values.

Regression Equation	R^2	Linearity range (µg/mL)	LOD (µg/mL)*	LOQ (ng/mL)*
y = 14561.65 (± 0.34)x - 7.71 (± 1.61)	0.99	2.5 - 100	0.37	1.11

The LOD and LOQ values were calculated according to the following equations (1) and (2):

$$C_{LOD} = 3.3\,\frac{\sigma_y}{b} \quad (1)$$

$$C_{LOQ} = 10\,\frac{\sigma_y}{b} \quad (2)$$

where C_{LOD} and C_{LOQ} are the sample concentrations corresponding to the LOD and LOQ, respectively, calculated as the standard deviation of the response (σ_y) on the slope of the calibration curve (b).

Table S2. Method validation: evaluation of precision (RSD %) and accuracy (Recovery %) in the short- and log-term period (intra-day and inter-day precision and accuracy values).

Theroetical Conc. (µg/mL)	Intra-day evaluation			Inter-day evaluation		
	Mean Conc. (µg/mL)	Precision (RSD%)	Accuracy (Recovery%)	Mean Conc. (µg/mL)	Precision (RSD%)	Accuracy (Recovery%)
40	0.040	0.299	99.60	0.039	1.04	98.68
	0.039	0.869	98.73			
	0.038	0.795	96.09			
80	0.080	0.105	100.31	0.08	0.83	99.56
	0.080	0.769	99.40			
	0.079	0.876	98.97			
Intra-day and Inter-day evaluation: analysis of 3 replicates of each external set within one day and for three consecutive days						

The HPLC method was validated in terms of precision and accuracy, in both the short- (intra-day) and the long-term (inter-day) period. For this purpose, an external set of two quercetin control solutions (with theoretical concentrations fixed at 40 µg/mL and 80 µg/mL, respectively), was run in triplicate on the same day and for three different days, respectively (Table S2). The mathematical model reported in Table S1 was then applied to calculate the concentration of the selected control solutions.

The method precision was calculated as the relative standard deviation (RSD %) among the concentration values achieved from consecutive injections, respectively within the short- or the long-term period (Table S2). The same experimental approach (that is control solution concentrations and replicates) proposed for the precision, was applied to evaluate the method accuracy expressed as percentage of quercetin recovery (Table S2).
